# The Arabic version of the modified-abbreviated math anxiety scale: Psychometric properties, gender differences, and associations with different forms of anxiety and math achievement

**DOI:** 10.3389/fpsyg.2022.919764

**Published:** 2023-01-05

**Authors:** Ahmed M. Megreya, Ahmed A. Al-Emadi, Ahmed A. Moustafa

**Affiliations:** ^1^Department of Psychological Sciences, College of Education, Qatar University, Doha, Qatar; ^2^School of Psychology, Faculty of Society and Design, Bond University, Gold Coast, QLD, Australia

**Keywords:** psychometric properties, math anxiety, test anxiety, science anxiety, general anxiety, math achievement

## Abstract

**Background:**

This study examined the psychometric properties (factor structure, measurement invariance, convergent and criterion validity, inter-correlations, and reliabilities) of an Arabic version of the modified-Abbreviated Math Anxiety Scale (m-AMAS) and gender differences in math anxiety in an Arabic speaking Middle Eastern country, Qatar.

**Methods:**

A large sample of students in grade 7 to 10 (*N* = 731) completed the m-AMAS, three different scales to measure science anxiety, test anxiety, and general anxiety, as well as a scholastic math achievement test.

**Results:**

The two-factor structure of the m-AMAS was confirmed, with good to adequate reliabilities, and its compositional measurement invariance was established across girls and boys in the four grades. In addition, math anxiety correlated positively with science anxiety, test anxiety, and general anxiety. Regression analyses showed that math anxiety was negatively associated with math achievement, even when test anxiety, science anxiety, and general anxiety were considered. Furthermore, girls showed higher math anxiety than boys.

**Conclusion:**

These adequate psychometric properties of the Arabic m-AMAS suggest that the construct of math anxiety has a cross-cultural similarity.

## Introduction

Recent research from a range of interdisciplinary scientific fields including educational psychology, clinical psychology, education, and cognitive neuroscience provide cumulative evidence that emotions greatly affect students’ learning and achievement (for an extensive review, see Pekrun and Linnenbrink-Garcia, 2014; Pekrun and Loderer, 2020). In specific, Pekrun (2014) has identified four groups of “academic emotions” that exert significant impact on learning, including achievement emotions (e.g., fear from failure), epistemic emotions activated by cognitive difficulties (e.g., frustration), topic-related emotions (e.g., math anxiety), and social emotions (e.g., social anxiety). The present study sought to investigate the construct of one of the most prevalent topic-related emotions, i.e., math anxiety, in an Arabic-speaking Middle Eastern country (Qatar).

### Math anxiety

Math anxiety refers to the feelings of fear, tension, and apprehension upon exposure to math-related materials during learning and assessment, which elicit a range of personal, educational, and cognitive consequences (Ashcraft, 2002). For example, individuals with high levels of math anxiety tend to avoid math subjects at school and university levels, consequently influencing their prospective career choices and performance in everyday life activities (Moustafa et al., 2020; Megreya and Al-Emadi, in press). According to the Organization for Economic Co-operation and Development’s report, the prevalence rate of math anxiety in 15-year old students is 30% (OECD, 2019). A large body of studies have shown that math anxiety is associated with a range of biological (e.g., genetic), educational (teaching and scholastic experiences), cognitive (e.g., working memory deficits), and neural factors (e.g., less activity in the frontal and parietal brain areas; Dowker et al., 2016). Although some studies suggest that math anxiety shares similar features with other forms of anxiety such as general anxiety (Hembree, 1990; O'Leary et al., 2017), test anxiety (Dew and Galassi, 1983; Hembree, 1990), and science anxiety (Henschel, 2021), others report that math anxiety is a separate and distinct construct (Vukovic et al., 2013; Carey et al., 2016, 2017; O'Leary et al., 2017). However, to the best or our knowledge, no previous study put together these different forms of anxiety in education to examine their differential associations with math anxiety. Therefore, the present study explores how gender, test anxiety, general anxiety, science anxiety, and math achievement predict math anxiety.

### Gender differences in math anxiety

Previous studies that examined gender differences in math anxiety showed mixed results (e.g., for a meta-analysis see Else-Quest et al., 2010). On one hand, several studies report higher math anxiety for girls in primary (Yüksel-Şahin, 2008; Griggs et al., 2013; Hill et al., 2016), secondary (Devine et al., 2012; Primi et al., 2014; Hill et al., 2016) and higher education (Dew and Galassi, 1983; Ferguson et al., 2015). On the other hand, other studies report no gender difference in primary (Ramirez et al., 2013; Ganley and McGraw, 2016) and secondary schools (Kyttälä and Björn, 2014). Gender stereotyping is considered a factor for increased math anxiety in girls (Steffens et al., 2010; Passolunghi et al., 2014). In addition, gender is found to moderate the relationship between math anxiety and math performance but results were inconsistent. For example, some studies found that math anxiety influences math performance more greatly in girls than in boys (Van Mier et al., 2018; Hart and Ganley, 2019). In contrast, in spite of the higher levels of math anxiety in early school-age girls, Szczygieł (2020) found that math anxiety impairs math performance more severely in boys. Therefore, the present study examines how math anxiety predicts math performance separately in boys and girls.

### Math anxiety and math performance

A large body of studies show that math anxiety and math performance influence each other, showing a negative relationship across different age groups (e.g., Barroso et al., 2021). Research has reported a range of moderators, such as gender, grade level, geographical region, and measurement, which influence the relationship between math anxiety and math performance (for a meta-analysis see Zhang et al., 2019). One of the most investigated moderator is cognitive processing, such that math anxiety negatively impacts working memory, which is in turn, critical for math performance (for a meta-analysis, see Finell et al., 2022). However, the causal direction of the negative relationship between math anxiety and math performance is highly debatable (Carey et al., 2016). On the one hand, the debilitating anxiety model posits that math anxiety negatively impacts math performance (Ashcraft, 2002). On the other hand, the deficit theory argues that poor math performance leads to math anxiety (Maloney et al., 2011). In a combination of these two approaches, a reciprocal theory suggests that both math anxiety and math performance interact and negatively influence one other (Carey et al., 2016; Zhang et al., 2019). Previous studies have provided mixed evidence supporting either the debilitating anxiety model or the deficit theory, rather than recommending the reciprocal approach (e.g., Carey et al., 2016). The present study sought to re-examine the relationship between math anxiety and math achievement when other forms of anxiety (test anxiety, science anxiety, and general anxiety) are controlled.

### Math anxiety measurement

Many math anxiety scales have been constructed (e.g., for reviews see Carey et al., 2016; Dowker et al., 2016) and one of the most recent measures is the Modified Abbreviated Math Anxiety Scale (m-AMAS; Carey et al., 2017). The m-AMAS was developed by revising the Abbreviated Math Anxiety Scale (AMAS; Hopko et al., 2003) to make it more suitable for British children (Carey et al., 2017). The AMAS involves 9 self-reported items measuring two subscales: Learning Math Anxiety (LMA, which consists of 5 items such as “Having to use the tables in the back of a math book”) and Math Evaluation Anxiety (MEA, which consists of 4 items such as “Thinking about an upcoming math test 1 day before”). In addition, a total score is calculated using the summation of all items. Using a 5-point Likert ranking scale ranging from 1 (low anxiety) to 5 (high anxiety), participants are required to indicate how anxious they would feel during certain situations in math class, as illustrated in those example items. Exploratory and confirmatory factor analyses supported this two-factor structure of the AMAS, with high internal consistency (αs = 0.90, 0.85, and 0.88) and test–retest reliability (*r*s = 0.85, 0.78, and.83) for the total score, LMA, and MEA, respectively (Hopko et al., 2003). The AMAS has been translated into a range of different languages such as Italian (Primi et al., 2014; Caviola et al., 2017), Polish (Cipora et al., 2015), Persian (Vahedi and Farrokhi, 2011), German (Schillinger et al., 2018), Spanish (Núñez-Peña et al., 2013), and Serbian (Sadiković et al., 2018). Across these translations, the two-factor model of the AMAS was replicated.

In the United Kingdom, Carey et al. (2017) made some modifications on the language and content of the AMAS. For example, the previously illustrated two items were modified as follows: “Having to complete a worksheet by yourself” and “Thinking about a math’s test the day before you take it” respectively. Carey et al. (2017) confirmed the two-factor solution of the m-AMAS, with good Alpha Cronbach reliabilities for the total score, LMA, and MEA (0.85, 0.77, and.79, respectively; Carey et al., 2017). The m-AMAS has been translated from English into several languages such as Serbian (Milovanović and Branovački, 2021) and Polish (Szczygieł, 2019). The two-factor structure of the m-AMAS and its adequate psychometric properties were replicated across these translations.

### The current study

The primary objective of this study was to examine the psychometric properties of an Arabic version of the m-AMAS by investigating construct validity (factorial structure and measurement invariance), inter-correlations between m-AMAS subscales, criterion validity (association with math performance), convergent validity (association with other forms of anxiety) and reliability. Secondary, this study aimed to examine gender differences in math anxiety. The two-factor structure of the Arabic version of the m-AMAS was expected (Hypothesis 1). In addition, we expected higher math anxiety in girls (Hypothesis 2) and strong positive correlations between math anxiety and other forms of anxiety in education (Hypothesis 3) but showing weaker partial correlations (Hypothesis 4).

## Materials and methods

### Participants

A total of 731 students (383 girls and 347 boys) in grades 7 to 10 in four government schools (two preparatory and two secondary) in Qatar volunteered to participate in this study. The sample size was estimated by power analysis, with an effect size of.25 (*p* = 0.05, with 0.95 power). Table 1 shows some characteristics of this sample. Data were collected with an approval from the Ministry of Education and Higher Education in Qatar and an approval of research ethics from a University Institutional Review Board (IRB) committee.

**Table 1:** Characteristics of participants.

Grade		Grade 7	Grade 8	Grade 9	Grade 10
Girls	N	82	102	106	94
	Age (mean and SD in years)	12.2 (0.5)	13.2 (0.5)	14.1 (0.5)	15.4 (0.7)
Boys	N	85	71	89	102
	Age	12.6 (0.7)	13.6 (0.8)	14.4 (0.8)	15.7 (0.7)

### Instruments

1. *The Modified-Abbreviated Math Anxiety Scale* (m-AMAS: Carey et al., 2017). The m-AMAS (Carey et al., 2017) was adapted from the AMAS (Hopko et al., 2003) to be more suitable for British children. It consists of 9 items measuring Learning Math Anxiety (LMA; 5 items) and Math Evaluation Anxiety (MEA (4 items). In addition, a total score represents the summation of these two factors. Using the original instructions of the scale (Carey et al., 2017), participants were asked to rate how anxious they would feel during some specified math-related situations such as “Thinking about an upcoming math test 1 day before” using a 5-point Likert ranking scale, ranging from 1 (low anxiety) to 5 (high anxiety).
2. *The Abbreviated Science Anxiety Scale* (*ASAS*; Megreya et al., 2021). Science anxiety refers to fearful emotions toward science learning (Bryant et al., 2013). The ASAS has been adapted from the m-AMAS (Carey et al., 2017) to measure science anxiety. Participants were asked to rate how anxious they would feel during some specified science-related situations such as “Thinking about an upcoming science test 1 day before” using a 5-point Likert ranking scale, ranging from 1 (low anxiety) to 5 (high anxiety). The ASAS consists of nine items, which belong to two main factors: Learning Science Anxiety (LSA, 5 items) and Science Evaluation Anxiety (SEA, 4 items). In addition, a total score represents the summation of these two factors. Megreya et al. (2021) confirmed this two-factor structure of the ASAS. In addition, Using samples of students from Grade 7 to 10, good to adequate reliabilities were reported for the three science anxiety scores that ranged from 0.87 to 0.89 for the total score, from 0.84 to 0.86 for LSA, and from .77 to .83 for SEA (Megreya et al., 2021).
3. *Test anxiety using the Brief FRIEDBEN Test Anxiety Scale* (von der Embse et al., 2013). Test anxiety refers to a combination of the cognitive, behavioral, and physiological symptoms of anxiety related to taking tests (von der Embse et al., 2018). This scale consists of 12 items measuring social derogation (5 items), cognitive obstruction (4 items), and physiological tenseness (3 items). Participants respond to each item using a six-point Likert ranking scale ranging from 1 (does not describe me at all) to 6 (describes me perfectly), with four reversed-score items. This three-factor structure was yielded through an exploratory factor analysis and was conformed using confirmatory factor analysis, with Alpha Cronbach reliabilities of 0.88, 0.86 and .81 for social derogation, cognitive obstruction, and physiological tenseness, respectively, (von der Embse et al., 2013). Consistently, across samples of students in Grades 7 to 12, McDonald’s ω reliability rates of the Arabic version of this scale were good to adequate ranging from .77 to .91 (Megreya et al., 2021).
4. *General Anxiety* (*Personality Inventory for DSM-5*: PID-5; Krueger et al., 2012). General anxiety was measured using the *PID-5* sub-scale of Anxiousness, which refers to “feelings of nervousness, tenseness, or panic in reaction to diverse situations; frequent worry about the negative effects of past unpleasant experiences and future negative possibilities; feeling fearful and apprehensive about uncertainty; expecting the worst to happen” (Krueger and Markon, 2014, p. 481). This sub-scale consists of nine items. Each item requires participants to describe themselves using a 4-point Likert-type scale (0 “*very false or often false,” 1 “Sometimes or Somewhat false,” 2 “Sometimes or Somewhat True,” and* 3 “*very true or often true”*), with only one reversed-score item, and the scores reflect the average of responses. An Arabic translation for the PID-5 has been validated in Qatar, with a Cronbach’s Alpha reliability of 0.89 for this subscale (Al-Attiyah et al., 2017; see also Megreya et al., 2021).
5. *Math* achievement. Math performance was measured using the students’ grades on math exams during the last semester before taking completing the questionnaires. Using specific blueprints clarifying the requirements of the exams, the scores and types of questions, and timing, these exams were developed by different school teachers, consisting of different numbers of math tasks (ranging from 20 to 30) and covering two units in each grade level. The vast majority of tasks (roughly 80%) required medium math skills.

### Translations and procedures

The m-AMAS (Carey et al., 2017) and test anxiety scale (von der Embse et al., 2013) were translated from English into Arabic with permission granted from the corresponding author of each questionnaire to the first author of the current study. Back-translation procedures were applied as following: (i) the scales were translated from English into Arabic; (ii) a professional translator, who had no prior experience with the scales, back-translated the Arabic versions into English; (iii) the back-translations were compared with the original scales by a native English researcher; (iv) modifications were made to produce the final Arabic translations of the scales.

Two schools were randomly selected from lists of preparatory and secondary schools in Doha, Qatar for each gender and then four classes were randomly assigned in each grade (from 7 to 10). All measures were administered to groups during class attendance, and administrations lasted for approximately 20 min. Data collection was done by two part-time research assistants (one female and one male), who were trained on the administration of each scale. Following IRB instructions, consent forms were required from the parents and students prior to participation in the study, particularly students were informed that they could withdraw at any time of the study without any consequences.

### Statistical procedures

Confirmatory Factor Analysis (CFA) was conducted using IBM Amos to examine the factor structure of the m-AMAS. For evaluating the model fit, we used the common fit indices, including Comparative Fit Index (CFI; >0.90), Tucker Lewis Index (>0.90), Root mean square error of approximation (RMSEA; <0.08), and Standardized Root Mean Square Residual (SRMR; <0.08). Using SmartPLS 4. Software (Ringle et al., 2022), compositional measurement invariance of the factorial structure was examined across the groups of gender and grades. Compositional invariance requires comparing the original composite score correlation (*c*) with the composite score correlation (*c*_*u*_). If *c* was equal or greater than 5.00% quantile of c_u_, then compositional invariance should be established (Henseler et al., 2016). McDonald’s Omega coefficient (ω) was used to examine the internal reliabilities of the m-AMAS and the other scales. In addition, Pearson correlation coefficients were used to examine the correlations among all variables. Furthermore, multiple linear regression analyses were conducted to examine a series of predictive models of math anxiety and math achievement from gender, test anxiety, general anxiety, science anxiety, and math achievement. Importantly, partial correlation coefficients were examined in order to control (taking away) the impacts of other variables (gender and other forms of anxiety) on the associations included in those regression models. To examine gender differences, a series of independent sample t-Tests were performed. For descriptives, we report means, standard deviations, and 95% confidence intervals.

## Results

### Factor structure

**Figure 1:**
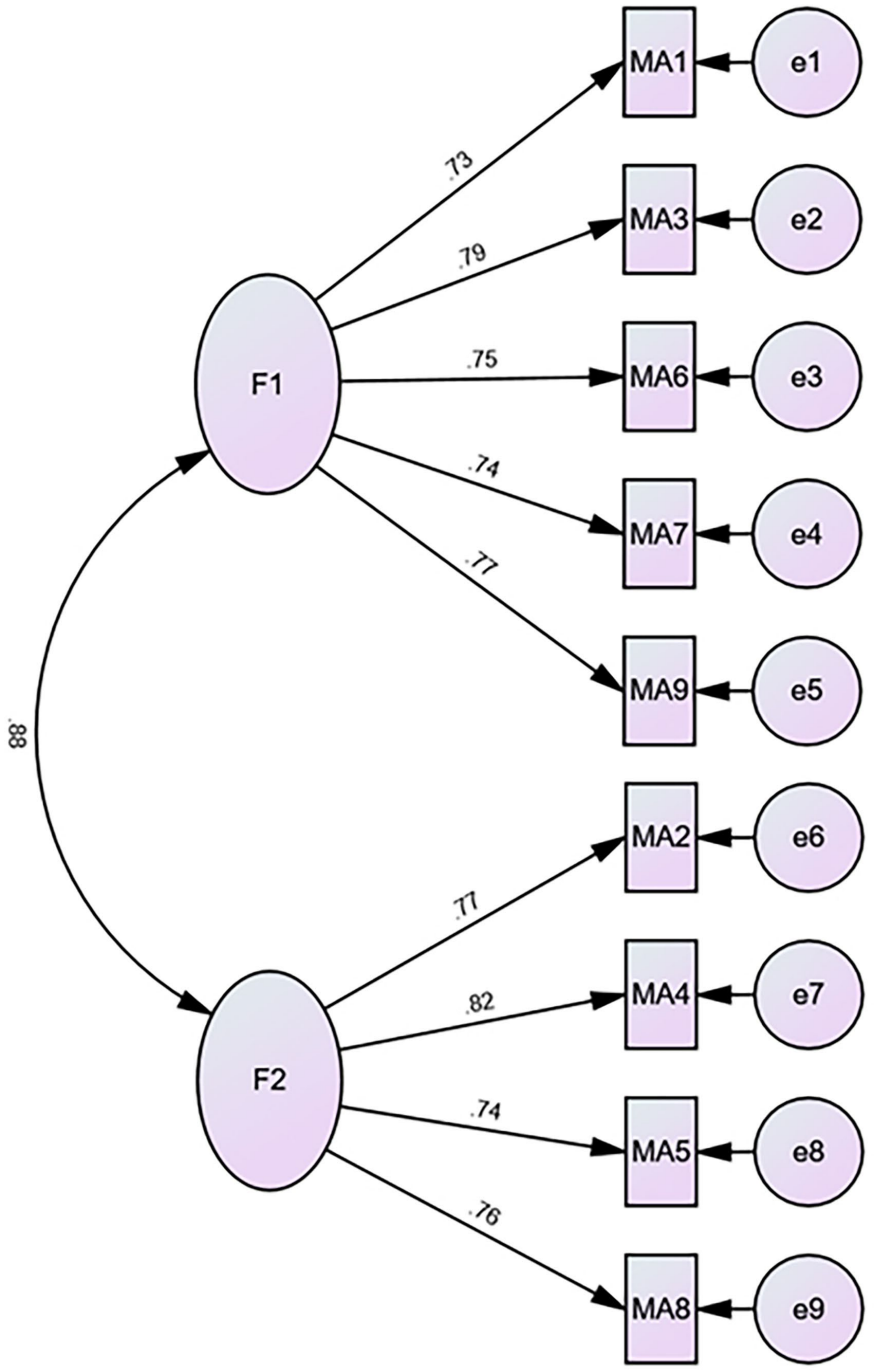
Factor structure of the Arabic version of the m-AMAS. F1 = Math learning anxiety; F2 = Evaluation math anxiety. The value on the arrow linking the two factors indicates Pearson’s correlation co-efficient while the values on the arrows directing to the items indicate standardized loadings, which are all acceptable (all ≥0.70).

CFA supported the two-factor model of the m-AMAS, χ^2^ (26) = 209.801, *p* ≤ 0.001. Fit indices reported good to generally acceptable fit: CFI = 0.950; TLI = 0.931; SRMR = 0.083; and RMSEA = 0.098. In addition, the loadings of the two factors with their corresponding items were generally acceptable, ranging from 0.73 to 0.79, with means of 0.76 and 0.72 for the LMA and MEA; respectively (see Figure 1).

### Measurement invariance

As presented in Table 2, compositional measurement invariance was established across all groups for the two subscales of the m-AMAS. However, for the MEA subscale, the original composite score correlation (*c =* 0.998) was just marginally smaller than the composite score correlation (*c*_***u***_ = 0.999), when a comparison was made between girls and boys in Grade 9.

**Table 2:** Compositional measurement invariance across girls and boys in grades 7 to 10.

	Girls vs. Boys/Grade 7		Girls vs. Boys/Grade 8		Girls vs. Boys/Grade 9	Girls vs. Boys/Grade 10		
	***c***	***c***_***u***_	***c***	***c***_***u***_	***c***	***c***_***u***_	***c***	***c***_***u***_
LMA	0.998	0.997	0.999	0.997	0.999	0.998	0.999	0.999
MEA	0.997	0.997	0.999	0.998	0.998	0.999	1	0.998

c = original composite score correlation; c_u_ = the composite score correlation. In order to establish measurement invariance, c should be equal or greater than c_u_.

### Internal reliability

Table 3 shows McDonald’s Omega coefficient (ω) of the scales, with 95% confidence Intervals (CIs) in parenthesis. Good to adequate reliabilities were obtained for the three math anxiety scores across the four grades that ranged from 0.90 to 0.93 (for the total score), from 0.86 to 0.90 (for LMA), and from 0.83 to 0.87 (for MEA).

**Table 3:** McDonald’s Omega coefficient (ω) (95% confidence Intervals).

	Grade 7	Grade 8	Grade 9	Grade 10
MA (9)	0.90 (0.88–0.92)	0.91 (0.89–0.93)	0.92 (0.90–0.94)	0.93 (0.92–0.94)
LMA (5)	0.86 (0.82–0.89)	0.85 (0.81–0.88)	0.87 (0.84–0.90)	0.90 (0.87–0.92)
MEA (4)	0.83 (0.79–0.87)	0.86 (0.83–0.89)	0.87 (0.84–0.90)	0.86 (0.83–0.89)
SA (9)	0.86 (0.83–0.89)	0.88 (0.85–0.90)	0.91 (0.89–0.93)	0.88 (0.86–0.91)
LSA (5)	0.82 (0.77–0.86)	0.84 (0.79–0.87)	0.89 (0.87–0.91)	0.84 (0.80–0.87)
SEA (4)	0.74 (0.67–0.80)	0.79 (0.73–0.84)	0.86 (0.83–0.89)	0.85 (0.81–0.88)
TA (12)	0.90 (0.88–0.92)	0.89 (0.86–0.91)	0.86 (0.83–0.93)	0.91 (0.89–0.93)
Social (5)	0.89 (0.87–0.92)	0.86 (0.83–0.89)	0.89 (0.86–0.91)	0.91 (0.88–0.93)
Cognitive (4)	0.88 (0.85–0.91)	0.89 (0.86–0.91)	0.90 (0.88–0.92)	0.88 (0.85–0.90)
Physiological (3)	0.85 (0.80–0.88)	0.79 (0.73–0.84)	0.85 (0.80–0.88)	0.88 (0.85–0.91)
GA (9)	0.82 (0.78–0.86)	0.81 (0.77–0.85)	0.84 (0.80–0.87)	0.87 (0.84–0.89)

MA = math anxiety; LMA = learning math anxiety; MEA = Math evaluation anxiety; SA = science anxiety; LSA = learning science anxiety; SEA = Science evaluation anxiety; TA = test anxiety; social = social derogation; cognitive = cognitive obstruction; Physio. = physiological tenseness; GA = general anxiety. Numbers of items of all measures are in parentheses.

### Convergent and criterion validity

Convergent validity was examined by the correlations between math anxiety and the other types of anxiety. In addition, consistent with previous studies (e.g., Megreya et al., 2021), concurrent-criterion validity, the strength of the correlation between a scale and an external test criterion (Cohen and Swerdlik, 2005), was investigated by the correlation between math anxiety and math performance. Table 4 shows the results. To summarize, math anxiety correlated positively with the other types of anxiety and correlated negatively with math achievement. These findings were robust as they were reported for the four groups of students from grade 7 to grade 10.

**Table 4:** The correlations between math anxiety and other forms of anxiety (science anxiety, test anxiety, and general anxiety) and math achievement.

	SA	LSA	SEA	TA	Social	Cognitive	Physio.	GA	Achievement
**All (N = 731)**									
MA	0.40** (0.46–0.34)	0.35** (0.42–0.29)	0.37** (0.43–0.31)	0.51^**^ (0.45–0.56)	0.45^**^ (0.38–0.51)	0.35^**^ (0.28–0.41)	0.40^**^ (0.34–0.46)	0.50^**^(0.43–0.56)	−0.41^**^ (−0.46 – -0.34)
LMA	0.37** (0.43–0.31)	0.35** (0.42–0.29)	0.32** (0.38–0.25)	0.48^**^(0.42–0.53)	0.42^**^ (0.35–0.49)	0.32^**^ (0.25–0.39)	0.38^**^ (0.32–0.44)	0.48^**^ (0.41–0.55)	−0.40^**^ (−0.46 – -0.34)
MEA	0.39** (0.45–0.32)	0.31** (0.38–0.25)	0.38** (0.44–0.32)	0.48^**^ (0.42–0.53)	0.42^**^ (0.36–0.48)	0.34^**^ (0.26–0.40)	0.37^**^ (0.31–0.44)	0.45^**^ (0.38–0.52)	−0.36^**^ (−0.42 – -0.29)
**Grade 7**									
MA	0.45** (0.56–0.32)	0.40** (0.52–0.27)	0.42** (0.53–0.28)	0.55^**^ (0.42–0.65)	0.49^**^ (0.35–0.60)	0.40^**^ (0.27–0.52)	0.44^**^ (0.31–0.57)	0.50^**^ (0.35–0.64)	−0.45^**^ (−0.56 – -0.31)
LMA	0.44** (0.55–0.31)	0.42** (0.54–0.29)	0.38** (0.51–0.24)	0.50^**^ (0.35–0.62)	0.45^**^ (0.30–0.57)	0.34^**^ (0.20–0.48)	0.43^**^ (0.29–0.55)	0.49^**^ (0.34–0.63)	−0.45^**^ (−0.57 – -0.31)
MEA	0.39** (0.51–0.26)	0.33** (0.45–0.18)	0.40** (0.52–0.27)	0.52^**^ (0.40–0.64)	0.46^**^ (0.33–0.58)	0.41^**^ (0.28–0.52)	0.40^**^ (0.27–0.53)	0.44^**^(0.29–0.57)	−0.37^**^ (−0.50 – -0.23)
**Grade 8**									
MA	0.37** (0.49–0.23)	0.35** (0.48–0.21)	0.30** (0.43–0.15)	0.51^**^ (0.39–0.62)	0.426^**^ (0.29–0.54)	0.38^**^ (0.23–0.50)	0.44^**^ (0.31–0.55)	0.45^**^ (0.31–0.58)	−0.383^**^ (−0.52 – -0.23)
LMA	0.30** (0.43–0.15)	0.32** (0.44–0.17)	0.21** (0.35–0.06)	0.45^**^ (0.32–0.57)	0.38^**^ (0.24–0.51)	0.31^**^ (0.16–0.45)	0.39^**^ (0.25–0.51)	0.44^**^ (0.30–0.57)	−0.35^**^ (−0.50 – -0.20)
MEA	0.40** (0.52–0.26)	0.35** (0.47–0.21)	0.35** (0.47–0.21)	0.51^**^(0.40–0.62)	0.41^**^(0.28–0.53)	0.40^**^ (0.26–0.52)	0.44^**^ (0.30–0.58)	0.40^**^ (0.26–0.54)	−0.36^**^ (−0.49 – -0.22)
**Grade 9**									
MA	0.45** (0.55–0.33)	0.39** (0.50–0.26)	0.43** (0.53–0.30)	0.50^**^ (0.37–0.59)	0.45^**^ (0.31–0.56)	0.30^**^ (0.17–0.41)	0.33^**^ (0.18–0.45)	0.53^**^ (0.40–0.65)	−0.37^**^ (−0.49 – -0.23)
LMA	0.44** (0.55–0.32)	0.39** (0.51–0.27)	0.41** (0.52–0.29)	0.48^**^ (0.35–0.58)	0.42^**^ (0.28–0.53)	0.30^**^ (0.17–0.41)	0.31^**^ (0.17–0.43)	0.51^**^ (0.39–0.63)	−0.37^**^ (−0.50 – -0.21)
MEA	0.40** (0.51–0.28)	0.34** (0.46–0.21)	0.39** (0.50–0.27)	0.46^**^ (0.33–0.56)	0.42^**^ (0.28–0.53)	0.26^**^ (0.12–0.39)	0.30^**^ (0.17–0.43)	0.49^**^ (0.36–0.62)	−0.33^**^ (−0.46 – -0.20)
**Grade 10**									
MA	0.36** (0.48–0.23)	0.29** (0.41–0.15)	0.37** (0.48–0.23)	0.50^**^ (0.40–0.59)	0.45^**^ (0.32–0.57)	0.34^**^ (0.23–0.46)	0.41^**^ (0.30–0.53)	0.50^**^ (0.36–0.62)	−0.44^**^ (−0.55 – -0.33)
LMA	0.32** (0.44–0.19)	0.29** (0.40–0.15)	0.30** (0.42–0.17)	0.49^**^ (0.39–0.59)	0.44^**^ (0.31–0.55)	0.35^**^ (0.23–0.46)	0.41^**^ (0.29–0.53)	0.48^**^ (0.36–0.60)	−0.43^**^ (−0.55 – -0.32)
MEA	0.36** (0.48–0.23)	0.26** (0.39–0.40)	0.40** (0.51–0.27)	0.44^**^ (0.34–0.54)	0.41^**^ (0.30–0.53)	0.30^**^ (0.18–0.41)	0.36^**^ (0.24–0.48)	0.47^**^ (0.33–0.59)	−0.40^**^ (−0.52 – -0.28)

All **p < 0.01; MA = math anxiety; LMA = learning math anxiety; MEA = math evaluation anxiety; SA = science anxiety; LSA = learning science anxiety; SEA = science evaluation anxiety TA = test anxiety; social = social derogation; cognitive = cognitive obstruction; Physio. = physiological tenseness; GA = general anxiety.

### Inter-correlations of the m-AMAS subscales

The LMA and MEA were positively correlated with each other in the four groups of students: Grade 7, *r* (165) = 0.74, *p* ≤ 0.001 (0.65–0.82, 95% CI); Grade 8, *r* (171) = 0.75, *p* ≤ 0.001 (0.68–0.83, 95% CI); Grade 9, *r* (193) = 0.78, *p* ≤ 0.001(0.72–0.83, 95% CI); Grade 10, *r* (194) = 0.79, *p* ≤ 0.001 (0.72–0.84, 95% CI).

### Multiple linear regression analysis

Multiple linear regression analyses were conducted to evaluate three models. Model 1 predicts math anxiety from gender, test anxiety, general anxiety, science anxiety, and math achievement using the whole sample. Model 2 predicts math achievement from math anxiety, test anxiety, science anxiety, and general anxiety in girls and boys separately. Model 3 predicts math achievement from learning math anxiety, math evaluation anxiety, test anxiety, science anxiety and general anxiety in girls and boys separately. The results showed that all of these models were statistically significant: Model 1, MA, *F* (5, 730) = 119.66, p ≤ 0.001, R^2^ = 0.452, LMA, *F* (5, 730) = 100.35, *p* ≤ 0.001, R^2^ = 0.409, and MEA, *F* (5, 730) = 95.97, *p* ≤ 0.001, R^2^ = 0.398; Model 2, girls, *F* (4, 383) = 41.76, *p* ≤ 0.001, R^2^ = 0.306, and boys, *F* (4, 346) = 26.80, *p* ≤ 0.001, R^2^ = 0.239; Model 3, girls, *F* (5, 383) = 32.90, *p* ≤ 0.001, R^2^ = 0.303, and boys, *F* (5, 346) = 20.49, *p* ≤ 0.001, R^2^ = 0.231. Table 5 shows the coefficients of these three models. To summarize, MA associated with math achievement in both girls and boys, even when test anxiety, science anxiety, and general anxiety were considered (see Model 2 partial correlations). In addition, both LMA and MEA predicted math achievement in both girls and boys, with higher influences for LMA than MEA (see standardized beta values in Model 3).

**Table 5:** The coefficients of the three regression models.

Models	Standardized Beta	*t* (*p*)	95% CI	Partial correlation
**Model 1**				
*MA*				
Gender	−0.091	−3.13 (*p* = 0.002)	−2.98 – −68	−0.115
TA	0.302	9.34 (*p* < 0.001)	0.17 – 0.26	0.328
GA	0.334	11.40 (*p* < 0.001)	3.96 – 5.61	0.390
SA	0.077	2.30 (*p* = 0.020)	0.01 – 0.16	0.085
Achive	−0.189	−6.01 (*p* < 0.001)	−0.20 – −0.10	−0.218
**LMA**				
Gender	−0.029	−0.95 (*p* = 0.344)	−1.01 – 0.35	−0.035
TA	0.292	8.71 (*p* < 0.001)	0.09 – 0.15	0.308
GA	0.333	10.95 (*p* < 0.001)	2.22 – 3,20	0.377
SA	0.060	1.73 (*p* = 0.085)	−0.01 – 0.08	0.064
Achive	−0.199	−6.08 (*p* < 0.001)	−0.12 – 0.06	−0.220
**MEA**				
Gender	−0.150	−4.95 (*p* < 0.001)	−2.09 – −0.91	−0.181
TA	0.274	8.09 (*p* < 0.001)	0.07 – 0.12	0.288
GA	0.292	9.51 (*p* < 0.001)	1.65 – 2.50	0.333
SA	0.087	2.64(*p* = 0.012)	0.01 – 0.09	0.091
Achive	−0.154	−4.46 (*p* < 0.001)	−0.09 – −0.03	−0.170
**Model 2**				
Girls				
TA	−0.060	−1.14 (*p* = 0.255)	−0.16 – 0.04	−0.058
GA	−0.094	−1.87 (*p* = 0.062)	−3.51 – 0.90	−0.096
SA	−0.338	−6.78 (*p* < 0.001)	−0.62 – −0.34	−0.329
MA	−0.234	−4.26 (*p* < 0.001)	−0.43 – −0.16	−0.214
Boys				
TA	−0.031	−0.56 (*p* = 0.575)	−0.13 – 0.07	−0.030
GA	−0.002	−0.03 (*p* = 0.972)	−1.93 – 1.86	−0.002
SA	−0.311	−5.87 (*p* < 0.001)	−0.66 – −0.33	−0.303
MA	−0.243	−3.94 (*p* < 0.001)	−0.49 – −0.16	−0.208
**Model 3**				
**Girls**				
TA	−0.097	−1.87 (*p* = 0.062)	−0.19 – 0.01	−0.096
GA	−0.138	−2.80 (*p* = 0.005)	−4.25 – −0.74	−0.143
SA	−0.085	−0.74 (*p* = 0.461)	−0.44 – 0.20	−0.038
LMA	−0.275	−0.2.50 (*p* = 0.013)	−1.18 – −0.14	−0.128
MEA	−0.166	−3.20 (*p* = 0.001)	−0.70 – −0.17	−0.163
**Boys**				
TA	−0.062	−1.11 (*p* = 0.269)	−0.16 – 0.04	−0.060
GA	−0.027	−0.52 (*p* = 0.600)	−2.37 – 1.37	−0.028
SA	−0.166	−1.39 (*p* = 0.167)	−0.64 – 0.11	−0.075
LMA	−0.168	−1.47 (*p* = 0.143)	−1.08 – 0.16	−0.079
MEA	−0.193	−3.22 (*p* = 0.001)	−0.86 – −0.21	−0.172

Data for gender factor was coded as 0 for girls and 1 for boys. Zero-order correlations refer to r (dependent, target variable), while partial correlations refer r (dependent, target variable) while partialling out the other variables.

### Gender differences

Table 6 shows gender differences in math anxiety scores. Girls had higher math anxiety than boys. This finding was consistently found for the four groups of students, but the difference in LMA between girls and boys in grade 10 was marginally significant (*p* = 0.07). Across all comparisons, Cohen’s d effect sizes were strong ranging from 0.26 to 0.71.

**Table 6:** Gender differences in math anxiety.

	Girls		Boys		*Skewness*	*Kurtosis*	*t*	*p*	Mean differences	Cohen’s *d*
	*M*	*SD*	*M*	*SD*						
**All (df = 729)**										
MA	24.8 (23.8–25.8)	10.4 (9.9–10.9)	19.7 (18.7–20.6)	9 (8.3–9.5)	0.533	−0.673	7.06	< 0.001	5.1 (3.7–6.5)	0.52 (0.37–0.67)
LMA	12.3 (11.7–13)	6.1 (5.7–6.4)	10.2 (9.6–10.7)	5.1 (4.7–5.4)	0.701	−0.538	5.08	< 0.001	2.1 (1.3–2.9)	0.38 (0.23–0.52)
MEA	12.5 (12–13)	5.1 (4.8–5.3)	9.5 (9–9.9)	4.4 (4.1–4.7)	0.262	−1.075	8.45	< 0.001	3 (2.3–3.7)	0.63 (0.48–0.77)
**Grade 7 (df = 165)**										
MA	24.4 (22.2–26.8)	10.9 (9.6–11.5)	20.3 (18.3–22.1)	9.1 (7.4–10.3)	0.618	−0.528	2.68	0.008	4.17 (1.1–7.2)	0.41 (0.11–0.72)
LMA	12.5 (11.2–13.9)	6.4 (5.6–7.1)	10.6 (9.4–11.7)	5.4 (4.5–6.1)	0.740	−0.481	2.08	0.039	1.91 (0.1–3.7)	0.32 (0.02–0.63)
MEA	11.9 (10.8–13.1)	5.3 (4.7–5.7)	9.7 (8.7–10.5)	4.3 (3.7–4.8)	0.340	−0.997	3.02	0.003	2.25 (0.8–3.7)	0.47 (0.16–0.77)
**Grade 8 (df = 171)**										
MA	25.4 (23.4–27.4)	10 (9–11)	18.8 (16.9–21)	8.6 (7–9.8)	0.399	−0.871	4.51	< 0.001	6.6 (3.7–9.5)	0.70 (0.38–1)
LMA	12.4 (11.2–13.6)	5.8 (5.2–6.3)	9.8 (8.8–11)	4.7 (3.9–5.4)	0.624	−0.705	3.08	0.002	2.6 (0.9–4.2)	0.48 (0.17–0.78)
MEA	13 (12–14)	5 (4.5–5.4)	9 (8.1–10)	4.4(3.6–4.9)	0.158	−1.194	5.50	< 0.001	4 (2.6–5.5)	0.84 (0.53–1.2)
**Grade 9 (df = 193)**										
MA	24.5 (22.7–26.4)	10 (8.8–11.1)	18.6 (17–20.3)	8.5 (7.1–9.6)	0.714	−0.283	4.44	< 0.001	6 (3.3–8.6)	0.64 (0.35–0.93)
LMA	12 (11–13.1)	5.8 (4.9–6.5)	9.3 (8.4–10.3)	4.6 (3.8–5.2)	1.011	0.299	3.52	0.001	2.7 (1.2–4.2)	0.51 (0.22–0.79)
MEA	12.5 (11.6–13.5)	5 (4.5–5.4)	9.2 (8.4–10.1)	4.3 (3.6–4.8)	0.318	−1.015	4.91	< 0.001	3.3 (2–4.6)	0.71 (0.41–1)
**Grade 10 (df = 193)**										
MA	24.6 (22.6–26.9)	10.9 (9.8–11.9)	20.7 (18.8–22.6)	9.4 (8.2–10.5)	0.420	−0.882	2.67	0.008	3.9 (1–6.8)	0.38 (0.10–0.66)
LMA	12.5 (11.3–13.8)	6.4 (5.8–6.9)	10.9 (9.9–12)	5.4 (4.7–6)	0.461	−1.006	1.82	0.07	1.5 (−0.12–3.2)	0.26 (−0.02–0.54)
MEA	12.2 (11.1–13.3)	5.1 (4.7–5.6)	9.8 (8.9–10.7)	4.5 (4–5)	0.237	−1.061	3.40	0.001	2.4 (1–3.7)	0.49 (0.20–77)

MA = math anxiety; LMA = learning math anxiety; MEA = Math evaluation anxiety.

## Discussion

Using a large sample of students from grade 7 to grade 10 in Qatar (N = 731), the present study examined the psychometric properties of an Arabic version of the m-AMAS (Carey et al., 2017) and gender differences. In support of Hypothesis 1, the CFA model fit indices confirmed the two-factor solution of the m-AMAS, although the RMSEA was larger than conventional recommendations. Compositional measurement invariances for these two factors were established between girls and boy in all of the four grades (except MEA in Grade 9). In addition, across different grades, the reliabilities of the total MA score and the two sub-scales (LMA and MEA) were good to adequate reliabilities (see Table 3). Given that the two-factor structure of the m-AMAS has been confirmed in different cultures including United Kingdom (Carey et al., 2017), Serbia (Milovanović and Branovački, 2021), Poland (Szczygieł, 2019), and Qatar (the present study), cross-cultural similarity of the nature of math anxiety construct is supported.

In support of Hypothesis 2, the present results showed that girls are more math anxious than boys in preparatory and secondary schools. This finding was consistent with previous studies (Devine et al., 2012; Primi et al., 2014; Hill et al., 2016). This gender difference seems to emerge early during development. A recent study by Szczygieł (2020) found that girls in grade 1 scored higher than boys in total math anxiety and math evaluation anxiety but not in math learning anxiety. In addition, Szczygieł (2020) found that general anxiety mediates the relationship between gender and math anxiety. Therefore, math anxiety may contribute to the refraining of females from STEM-related subjects and careers. However, Halpern et al. (2007) suggested that there is no “single” factor for these gender differences in math achievement and STEM careers, but many factors including early experiences, biological constraints, educational policies, and socio-cultural contexts could influence and rather interact in complex ways.

In support of Hypothesis 3, the present study showed that math anxiety positively correlated with science anxiety, test anxiety, and general anxiety (see Table 4). This finding supported the suggestion that math anxiety shares some similar features with general anxiety (e.g., Ashcraft, 2002), test anxiety (e.g., Hembree, 1990), and science anxiety (Henschel, 2021). Importantly however, in support of Hypothesis 4, the present results showed modest or weak partial correlations between m-AMAS scores and these different forms of anxiety when gender, general anxiety, test anxiety, science anxiety, and math achievement were controlled (see Table 5). These week partial correlations replicated the results of Hill et al. (2016). Together, these results confirmed the suggestion that math anxiety is a distinct construct (Vukovic et al., 2013; Carey et al., 2016, 2017; O'Leary et al., 2017). Using this same approach, Megreya et al. (2021) suggested that science anxiety is another distinct construct.

Replicating many previous studies (e.g., for a review see Carey et al., 2016), the results of the present study showed that math anxiety negatively correlated with math achievement (Table 4). Although the negative correlation between math anxiety and math performance has been replicated in many countries, some cross-cultural variations were noticed. For example, the impact of math anxiety on math performance was stronger in Western countries (such as New Zealand and Norway) than Eastern countries (such as Japan, Korea, and Thailand; OECD, 2019). Future work should investigate why math anxiety does not sometimes influence math performance.

From a cultural perspective, the present study contributes novel data to math anxiety literature from an Arabic-speaking Middle Eastern culture. In fact, the vast majority of psychological studies have been conducted with participants from Western, educated, industrialized, rich and democratic (WEIRD) societies (mostly Americans; see Henrich et al., 2010a,b). Similarly, most knowledge of math anxiety has come from such WEIRD countries (e.g., for reviews see Dowker et al., 2016). Importantly, however, previous studies that have used PISA data reported both cross-cultural similarities and variations for math anxiety among different cultures (Lee, 2009; Fan et al., 2019). For example, Fan et al. (2019) identified three math anxiety profiles (low, mid, and high) among 15-year old students from Finland, Korea, and the United States and found that the percentages of students in each profile differed across these nations, with United States having the highest prevalence of High MA and Finland the lowest. In addition, Lee (2009) found that math high achieving Asian countries (such as Korea and Japan) showed low math self-concept and high math anxiety in spite of their high scores on math performance, while some of the Western European countries (such as Finland, Netherlands, Liechtenstein, and Switzerland) demonstrated high math performance and low levels of math anxiety. Therefore, in order to explore the factors underlying these cross-cultural differences in math anxiety, more studies are needed outside those few WEIRD countries. The present study is an attempt to fill in this gap.

The present study carries some important implications. Namely, the Arabic version of the m-AMAS could be reliably utilized to assess math anxiety in Arabic-speaking students in Middle Eastern countries or those who are currently refugees in Western nations. Any intervention would not be possible unless an accurate assessment is provided using a reliable measure with adequate psychometric proprieties. This is particularly important for high school students who might have a broader repertoire of negative experiences in math (e.g., for a review see Ashcraft et al., 2007), especially because high levels of math anxiety was found to be a critical factor for avoiding STEM-related courses and careers (Megreya and Al-Emadi, in press).

This study is not without limitations. First, all instruments used here were self-reported scales, except the math achievement test. Second, given that all subjects in the current study were from grades 7 to 10, the present findings may not be generalizable to other grades. Third, the math achievement data, which were collected using a school math exam had clear ceiling and roof effects. Finally, although measurement invariance was established across all groups for the two m-AMAS subscales, there was a minor measurement non-invariance between girls and boys in a specific subscale (MEA) and a specific grade (Grade 9). Accordingly, future work needs to replicate the present results in other populations such as primary school students and using behavioural measures of math anxiety. In addition, future research may be favourable to include a standardized math performance test. Furthermore, regarding the specific minor measurement non-invariance, this initial finding needs a replication before any conclusion could be made. If it is replicated, alternatively we would need to examine partial invariance and comparing the differences of path coefficient estimates between structural equation models (Pohl et al., 2021). Nevertheless, the present study replicated the adequate psychometric properties of the m-AMAS, gender differences in math anxiety, and the uniqueness of math anxiety as compared with other forms of anxiety (science, test, and general anxieties) in a highly neglected population in psychological literature, Middle Eastern Arabic culture, suggesting a cross-cultural similarity of the math anxiety construct.

## Data availability statement

The original contributions presented in the study are included in the article/Supplementary material, further inquiries can be directed to the corresponding author.

## Ethics statement

This study involves human participants and it was reviewed and approved by Qatar University IRB Committee (Qatar University, Qatar). Written informed consent to participate in this study was provided by the participants and their parents

## Author contributions

AMM, AA-E, and AAM contributed to design of the study. AMM collected the data, performed all statistical analyses, wrote the first draft of the manuscript, and addressed all reviewers’ comments. AA-E helped in data collection and writing the first draft. AAM contributed in writing some sections of the introduction in an early manuscript. All authors contributed to the article and approved the submitted version.

## Funding

This study was made possible by an NPRP-C # Subproject (NPRP12C-33955-SP-90), which is a part of an NPRP-C project (NPRP12C-0828-190023) from the Qatar National Research Fund (a member of Qatar Foundation). The statements made herein are solely the responsibility of the authors.

## Conflict of interest

The authors declare that the research was conducted in the absence of any commercial or financial relationships that could be construed as a potential conflict of interest.

## Publisher’s note

All claims expressed in this article are solely those of the authors and do not necessarily represent those of their affiliated organizations, or those of the publisher, the editors and the reviewers. Any product that may be evaluated in this article, or claim that may be made by its manufacturer, is not guaranteed or endorsed by the publisher.

## Supplementary material

The Supplementary Material for this article can be found online at: https://www.frontiersin.org/articles/10.3389/fpsyg.2022.919764/full#supplementary-material
